# Updates on Clinical Use of Liquid Biopsy in Colorectal Cancer Screening, Diagnosis, Follow-Up, and Treatment Guidance

**DOI:** 10.3389/fcell.2021.660924

**Published:** 2021-05-24

**Authors:** Omayma Mazouji, Abdelhak Ouhajjou, Roberto Incitti, Hicham Mansour

**Affiliations:** ^1^GES-LCM2E, FPN, Mohamed First University, Oujda, Morocco; ^2^Al-Azhar Oncology Center, Rabat, Morocco; ^3^Computational Bioscience Research Center (CBRC), King Abdullah University of Science and Technology (KAUST), Thuwal, Saudi Arabia

**Keywords:** colorectal cancer, liquid biopsy, biomarker, screening, diagnosis, prognosis, targeted therapy, circulating tumor DNA

## Abstract

Colorectal cancer (CRC) is one of the most common cancers worldwide, being the third most diagnosed in the world and the second deadliest. Solid biopsy provides an essential guide for the clinical management of patients with colorectal cancer; however, this method presents several limitations, in particular invasiveness, and cannot be used repeatedly. Recently, clinical research directed toward the use of liquid biopsy, as an alternative tool to solid biopsy, showed significant promise in several CRC clinical applications, as (1) detect CRC patients at early stage, (2) make treatment decision, (3) monitor treatment response, (4) predict relapses and metastases, (5) unravel tumor heterogeneity, and (6) detect minimal residual disease. The purpose of this short review is to describe the concept, the characteristics, the genetic components, and the technologies used in liquid biopsy in the context of the management of colorectal cancer, and finally we reviewed gene alterations, recently described in the literature, as promising potential biomarkers that may be specifically used in liquid biopsy tests.

## Introduction

The World Health Organization projected that, in the next few years, cancer will kill more than a dozen million people every year worldwide. The figures exceed those of all other death causes, such as infection, cardiovascular diseases, traffic accidents, and other registered so far (World Health Organization, 2020b). The global prevalence of cancer is predicted to increase by 75% by 2030 (Ferlay et al., 2019; World Health Organization, 2020b). This rise is projected to be significantly larger in developing countries and could reach 90% in the poorest countries (Ferlay et al., 2019; World Health Organization, 2020b). Despite the advances in diagnostic tools and treatments, colorectal cancer (CRC) remains one of the leading causes of cancer mortality worldwide, accounting for more than 9% of all cancer deaths (Boyle and Langman, 2000; Ferlay et al., 2019). According to a recent report from the GLOBOCAN database, the second highest mortality rates among cancers were observed for CRC, with an estimated number of 935,173 deaths (both sexes and all ages; Ferlay et al., 2019; World Health Organization, 2020a). Prognosis for CRC depends on several parameters, among which the two most important are age and stage at diagnosis.

Several large studies have shown that the risk of developing CRC begins to increase noticeably after the age of 50. After that, the risk continues to double approximately with each succeeding decade (Haggar and Boushey, 2009). Although young age is associated with more advanced disease stage and unfavorable tumor characteristics, young age was shown to be a prognostic factor for better survival (Li et al., 2014). Less advanced stage I tumors are associated with a higher 5-year survival amounting to more than 80%, as compared to more advanced tumors like stage IV, which is associated with a 5-year survival rate of less than 5% (O’Connell et al., 2004; Noone et al., 2018). Unfortunately, most CRC cases are diagnosed at advanced stages, when curative surgical treatment is not sufficient, and chemotherapy or targeted therapy must be used. In contrast, CRC is preventable and curable when diagnosed early (Hamzehzadeh et al., 2017). These statistics show how crucial it would be to have screening tools to accurately detect CRC tumors at the early stage, enabling more successful fighting CRC and reduction of mortality caused by this cancer. In diagnosis, the golden standard for the determination of CRC status is the pathological analysis of tissue biopsy. Obtaining such biopsy requires relatively costly and complex medical devices as colonoscopy or flexible sigmoidoscopy, and well-trained staff (Baxter and Rabeneck, 2009). Nevertheless, biopsy has several important limitations: it is invasive, is expensive, is uncomfortable, is difficult to be performed in serial tests, has technical limitations associated with tumor location, and is not efficient in targeting tumor cells subpopulations (Fernández-Lázaro et al., 2020). Tissue extraction carries also risks, and it is inaccessible for some cases (Baxter and Rabeneck, 2009). Moreover, it gives tumor picture only at a single location, and it could ignore spatial tumor genetic heterogeneity. Therefore, it cannot be used in screening programs to detect CRC at early stage and it is not suitable for longitudinal monitoring (Baxter and Rabeneck, 2009; Hamzehzadeh et al., 2017; Fernández-Lázaro et al., 2020). In contrast to tissue biopsy, liquid biopsy has several advantages in CRC management. In this review, we describe the concept of liquid biopsy and its applications in the management of colorectal cancer patients.

## Liquid Biopsy Background

The concept of liquid biopsy is that of searching circulating biomarkers to detect tumor traces released from primary tumor and/or metastasis sites (Peng et al., 2017; Fernández-Lázaro et al., 2020). The extensive clinical research conducted to date clearly showed that liquid biopsy can provide information on the molecular status of CRC at any disease stage of the tumor, whether primary or metastasis stage (Wang et al., 2019), and in a comprehensive way which could strongly help oncologists in treatment guidance, particularly in identifying abnormalities leading to cancer initiation, to make treatment decisions and to monitor patient response to treatment (Cheung et al., 2018; Cortés-Hernández et al., 2020; Fernández-Lázaro et al., 2020).

In the field of noninvasive genetic tests, the first study that provided a conceptual framework and a practical basis for a new molecular approach to detect CRC was related to the development of the first assays based on the detection of the presence of gene mutations in DNA extracted from the stool (Sidransky et al., 1992; Mansour, 2014). This finding raised considerable hope of a possibility of developing a noninvasive genetic test using stool of CRC patients. Subsequent work assessed several biomarkers in stool and in other body fluids (Ebert et al., 2006; Roperch et al., 2013; Amiot et al., 2014; Mansour, 2014). Using information from detection of genetic anomalies affords a sensitivity (i.e., the ability to avoid individuals with CRC being falsely diagnosed as healthy) which can be substantially high, while still maintaining reasonably high specificity (Ebert et al., 2006; Roperch et al., 2013; Amiot et al., 2014; van Lanschot et al., 2017). The use of this molecular approach was also tentatively extended to post-therapeutic surveillance (Imperiale et al., 2014).

Molecular assays for CRC detection currently in use or under development are based on assessing genetic and/or epigenetic anomalies or a combination (Palmirotta et al., 2018). While other type of markers exists, such as proteins or RNA biomarkers, they might not be suitable for routine tests, because, before performing detection experiments, these assays need special patient preparations, stabilizing buffers, and adequate temperature for sample storage. The process is long, is error-prone, and may be a barrier for patients’ adherence to the CRC detection and monitoring protocols. Unlike protein or RNA-based assays, genetic assays are based on a more streamlined analytical experiment. In addition to that, genetic assays depend much less on setting varying thresholds on relevant experimental parameters when specified for distinguishing cancer patients from the controls (Robertson and Imperiale, 2015). Moreover, many genetic biomarkers can be combined in a single assay, which is more challenging with protein-based assays (this is not a problem with RNA-based ones). In addition to that, the large development of massively parallel sequencing tools and the evolution of our knowledge and understanding of genetics show that genetic variations and epigenetic modifications can strongly impact screening, diagnosis, and prognosis options of CRC-affected patients, as well as how patients may respond to specific therapy. Several noninvasive epigenetic and genetic tests are now available, having high sensitivity and specificity and being low-risk, cost-effective, and easy to implement in clinical settings or across a large population for CRC patient screening (Ebert et al., 2006; Mansour et al., 2012; Roperch et al., 2013).

## Significance of Liquid Biopsy

The extensive clinical research progress and advancements that have been made since the human genome was first sequenced have greatly increased our knowledge on cancer genetics and have participated in the development of new cutting-edge molecular biology tools. Several genetic and epigenetic alterations have been shown to initiate and sustain specific deregulated cellular signaling pathways involved in CRC tumors. Today, these alterations can be easily detected in body fluids without the need to investigate the initial tumor site. From a simple blood collection, we can extract circulating tumor cells (CTC), circulating tumor DNAs (ctDNAs), and/or other extracellular vesicles (EV) and monitor molecular alterations in CRC patients. This procedure can be frequently repeated over time in order to monitor changes that occur during treatment, serving as an early indicator of tumor recurrence, drug resistance, or metastasis with a view to adapting, escalating, or changing the treatment strategy.

Liquid biopsy has several advantages: it is a noninvasive, fast, and easy tool to perform. In terms of noninvasiveness, sampling body fluids (blood or stool) does not require sophisticated equipment or highly skilled human intervention (Neumann et al., 2018; Fernández-Lázaro et al., 2020). For instance, some companies provide kits to individuals who can take the stool samples at home, thus speeding up the clinical process. The result can be obtained in a few hours, as compared to tissue extraction and pathological analysis, which can take several days. Moreover, liquid biopsy generates less morbidity than the conventional method due to its minimally invasive or noninvasive nature. Performing a series of liquid biopsies instead of solid biopsies avoids unnecessary health risks for the patient (Vymetalkova et al., 2018; Fernández-Lázaro et al., 2020), such as hyper-vascularized tumor rupture leading to catastrophic bleeding and hemorrhage risk and tumor seeding, regarding which several studies have shown that through aspiration cytology and biopsy extraction, many patients developed cancer at multiple sites (Shyamala et al., 2014).

CRC is known for its wide temporal and spatial intratumor heterogeneity; there are three major molecular pathways that produce this heterogeneity: genomic instability, microsatellite instability, and CpG island methylator phenotype (Dang et al., 2020). Detecting this heterogeneity through an invasive solid biopsy represents a great challenge, while this could be possible in some cases through a liquid biopsy (Vacante et al., 2020).

## Liquid Biopsy Components

**Figure 1:**
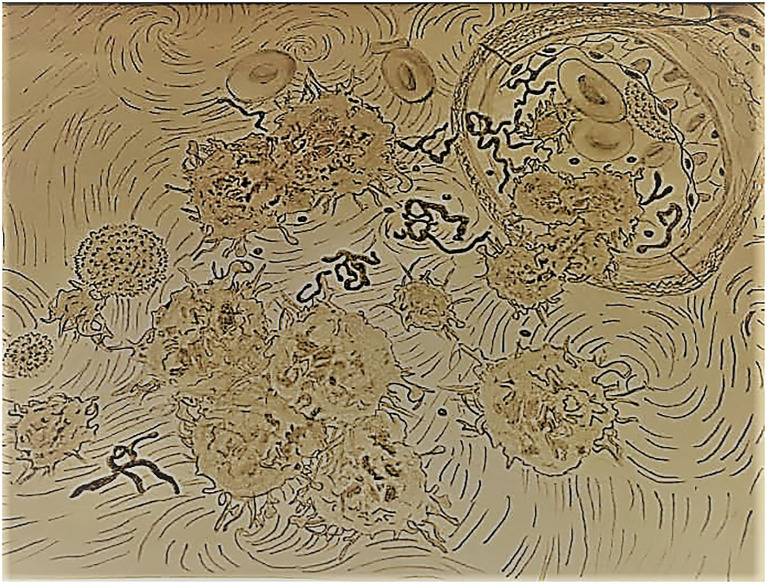
The main types of tumor traces targeted in liquid biopsy (circulating tumor cells, circulating tumor exosomes, circulating tumor DNA, circulating tumor RNA). The molecular biology tools routinely used are flow cytometry, real-time-PCR, next-generation sequencing (NGS), microarray, and BEAMing technology.

The main types of tumor traces targeted in liquid biopsy are CTC, circulating tumor exosomes, ctDNAs, and circulating tumor RNAs (ctRNAs; Heitzer et al., 2015; Jia et al., 2017; Vymetalkova et al., 2018; Ding et al., 2020; Eslami-S et al., 2020; Figure 1).

Circulating tumor cells are cells released into the bloodstream from the primary or metastatic high-dividing tumor cells (Shen et al., 2017; Muinelo-Romay et al., 2018). CTCs are the first tumor biomolecules discovered in 1869 by Ashworth in the blood of a deceased patient with metastatic cancer. CTCs exist in various forms: as single cells with several epithelio-mesenchyme transition (EMT) phenotypes or as clusters bound to platelets, macrophages, and/or reactivated stromal cells (Satelli et al., 2015). CTCs are present in a few cells to hundreds per milliliter of whole blood; in addition, CTCs are larger than white blood cells and measure between 15 and 25 μm (Yang et al., 2019). Several groups have been working on CTC detection methods and have developed a variety of methods to overcome technical challenges to efficiently capture CTCs in the blood (Shen et al., 2017; Yang et al., 2019). The application of these tools in gastrointestinal malignancies was promising for early diagnosis, treatment planning, prognostic stratification, and metastasis monitoring (Pantel and Alix-Panabieres, 2017; Yang et al., 2019).

Exosomes are EV and homogeneous particles, stable and easily detectable in terms of their size. Exosomes are detected in almost all body fluids and express specific markers, such as HSP70 and ALIX, that distinguish exosomes from other subcellular vesicles (Halvaei et al., 2018; Zhou et al., 2020). Exosomes can be considered as diagnostic and prognostic biomarkers in cancer. Many commercial kits provide rapid and efficient separation of exosomes from a small amount of human body fluid. In the case of CRC, the ExoScreen technique detects circulating exosomes in patients’ blood samples (Fernández-Lázaro et al., 2020).

Circulating tumor DNAs are DNA fragments found in the bloodstream following different events, such as apoptosis, necrosis, and macrophage digestion. Some of them derive from malignant tumor cells, thus providing information about specific mutations in cancer (Takeda et al., 2019). In 1948, Mandel et al. were the first who hinted at the occurrence of circulating free-DNA (cfDNA) in plasma from different diseases. Despite this early discovery, the concept of liquid biopsy using ctDNA did not start being used until 1977, when other researchers identified the presence of ctDNAs in the body fluid of patients with cancer (Aghamir et al., 2020). The size range of ctDNAs varies between 150 and 10,000 bp, but the vast majority of ctDNAs are found with a size of 166 bp, like nucleosomes. Possibly, ctDNAs might be released during cell apoptosis, necrosis, or following cell lysis by immune cells (Shen et al., 2017). In CRC, a high degree of concordance between somatic mutations detected in tumor tissue and those in ctDNAs was described in blood samples from patients with early or advanced tumors, which means that the ctDNAs retain the same genetic signatures as those present in tumor tissue (Vidal et al., 2017; Li et al., 2019). The sheer amount of ctDNA found in peripheral blood differs between normal individuals and those with colorectal cancer. Indeed, circulating tumor DNA levels are higher in cancer patients than in normal individuals. The number of mutant DNA fragments can range between 1 and 1800 fragments per milliliter of plasma (Diehl et al., 2008). This quantity is low in patients with early-stage CRC, while in the case of metastatic CRC, the quantity can largely exceed these values (Shen et al., 2017). These quantities are present as a small fraction of the total cfDNA, ranging from 0.01 to 50% depending on tumor stage (Diehl et al., 2008). It is also important to mention that the information gathered from ctDNA varies depending on tumor type, since not all tumors release ctDNA. Moreover, ctDNA in cancer patients occurs in low amounts in blood compared to cfDNA; this is due to several factors such as cancer stage, tumor vascularization, tumor burden, metastasis event, and rates of cell necrosis and apoptosis.

Circulating RNAs. In addition to ctDNAs and CTCs, free ctRNAs can be used as noninvasive tumor markers in colorectal cancer. The denomination of ctRNA includes several RNA types, as micro-RNA (miRNA), other noncoding RNA (ncRNA), and messenger RNA (mRNA; Fernández-Lázaro et al., 2020). miRNAs are the most studied over the past decade and may be the most widely described noninvasive biomarkers in colorectal cancer (Table S1). They are detected in both serum and fecal samples and have attracted more attention, due to their stability and resistance to RNase-mediated degradation (Toiyama et al., 1870). They can be released into the bloodstream in two forms, either in association with RNA binding proteins or packaged in exosomes, both providing protection and stability of RNAs in body fluids (Shigeyasu et al., 2017; Fernández-Lázaro et al., 2020).

Significant research effort has been focused on the detection of ctDNAs in cancer and provides an overview of the detection performance across cancer types, experimental conditions, and clinical practice (Vymetalkova et al., 2018). Regarding CRC, clinical research studies have shown that information gathered by ctDNAs can afford good sensitivity, specificity, and predictive power both in the diagnosis and in the follow-up of CRC patients (Mansour, 2014). There is now large evidence that liquid biopsy represents an essential tool in oncology, through the collection of samples of minimally invasive fluids. Targeting ctDNAs or other cell components in liquid biopsy is an appropriate method to detect genetic abnormalities harbored by tumor cells. In this context, liquid biopsy can detect tumor heterogeneity, monitor molecular changes during and after treatment (Diehl et al., 2008; Toledo et al., 2017; Trojan et al., 2017), and allow to set up a molecular profile on the tumor burden in patients with colorectal cancer, as well as with other types of cancers (Arneth, 2018; Cheung et al., 2018; Tadimety et al., 2018). In clinical practice, liquid biopsy has several advantages in filling the gaps generated by conventional methods, in particular solid biopsy, in that it can allow (1) the early detection of mutations, and in particular “actionable” (i.e., therapeutically targetable) mutations, and (2) avoiding the use of therapies associated with tumor resistance.

## Molecular Biology Approaches in Liquid Biopsy

To date, several technologies based on the molecular biology of ctDNAs or DNA/RNAs from CTC or EV have been adapted for achieving ctDNAs identification, and analysis (Figure 1). They can be classified into two approaches: massively parallel sequencing technologies or next-generation sequencing (NGS) and digital genomic techniques such as digital PCR (dPCR). NGS can target any gene or regulatory region in ctDNA by selecting genomic regions of the target of interest, before sequencing and during the library preparation. This approach assures the analysis of large parts of the genome and allows the identification of several types of genetic alterations with high technical sensitivity (Osumi et al., 2019).

In case of presence of rare alleles and limited amount of input DNA material, the sensitivity of ctDNA detection can be further improved by using dPCR (Osumi et al., 2019). This approach provides increased identification and absolute quantification of ctDNA by dividing samples into multiple independent quantitative PCR reactions. The reactions performed contain ideally one target sequence, or few targets or none, so that the total concentration of the target sequence is obtained by a fraction of the positive amplification partitions. This partitioning of samples ensures a better detection of genetic abnormalities, including rare mutations that are difficult to detect (Quan et al., 2018; Chin et al., 2019). Digital genomic technologies offer higher technical sensitivity compared to most massively parallel sequencing technologies and have been used as an orthogonal method for validation of results and for the quantification of ctDNA. The dPCR can identify rare frequent alleles with high accuracy (Cheung et al., 2018). The NGS can also detect rare mutations but with a lower technical sensitivity than dPCR, unless (potentially costly) high sequencing depth is reached (Cheung et al., 2018; Mansour et al., 2020). In contrast to dPCR, massively parallel sequencing approaches can be designed to detect somatic mutations in either known or unknown driver genes. Besides these methods, there are other technologies allowing precise detection of ctDNA at high sensitivity, such as digital droplet PCR (ddPCR), BEAMing technology, and microarray (García-Foncillas et al., 2017; Wu et al., 2020). In Table 1, we give a brief description of these techniques highlighting their limitations.

**Table 1:** The limitations of techniques routinely used for the detection of ctDNA or CTC.

Method	Description	Limits	References
Digital PCR (dPCR)	dPCR ensures increased identification and absolute quantification of circulating tumor DNA by amplification of the target in the presence of fluorescent dye.	The dPCR’s multiplexing (simultaneous assay of several targets) is limited. Unable to detect all alteration types that could occur within a single target. Needs protocol optimization for each alteration type. Unable to simultaneously detect gene expression and gene alteration. Detect only known mutations.	Mansour et al., 2012; Fernández-Lázaro et al., 2020
Next-generation-sequencing (NGS)	NGS allows analysis of large parts of the genome and can identify multiple mutations with increased sensitivity.	Library and sequencing kits are expensive and time-consuming. Sample procession and library preparation need experienced specialists. Primary and secondary data analysis require bioinformatics expertise. Raw data can be noisy. Need adequate sequencing coverage. Can miss low-frequency variants. Need orthogonal validation.	Noone et al., 2018; Wang et al., 2019; Fernández-Lázaro et al., 2020
Droplet digital PCR (ddPCR)	DdPCR represents a highly sensitive and absolute quantification strategy that allows detection of low-frequency variants by amplification of single DNA molecules. This strategy is based on splitting the sample into several individual droplets and each droplet undergoes a PCR reaction that will then be analyzed to determine the positive fractions in the original sample.	Presence of technical false negative and false positive results. Lack of full automation of the technique. Detection of only known mutations.	Peng et al., 2017; Wang et al., 2019; Fernández-Lázaro et al., 2020
BEAMing technology (beads, emulsion, amplification, and magnetics)	BEAMing allows the detection of ctDNAs based on the amplification of the DNA segment by ePCR, then identification and quantification of the beads containing the mutation by flow cytometry.	Technical complexity difficult to tackle for routine analysis. The success rate of emulsion PCR is low. Targets only a small number of alterations. Detection only known mutations. Introduction of biases due to requirement of pre-amplification of the target sequence.	Cheung et al., 2018; Wang et al., 2019; Fernández-Lázaro et al., 2020
CELLTRACKS® AUTOPREP® System and CELLTRACKS ANALYZER II® System (CellSearch Kit)	CellSearch is based on the selection of circulating epithelial cells from peripheral blood samples by the use of magnetic beads and under the influence of a magnetic field.	CTCs with mesenchymal features cannot be detected. Only CTCs that retain epithelial features can be detected. The test requires the collection of large volume of blood for analysis. Only few cells are detected.	Neumann et al., 2018; Noone et al., 2018; Cortés-Hernández et al., 2020; World Health Organization, 2020a

A number of technologies based on the biological or physical properties of CTCs have been developed for CTC isolation and identification (Shen et al., 2017; Yang et al., 2019). CTCs are detected by an immunomagnetic separation technology based on the combination of surface antigens with magnetic beads attached to specific antibodies. For example, an antibody against the epithelial cell adhesion molecule (EpCAM) is used, then the antigen-antibody complex formed is separated under the effect of a magnetic field (Cheung et al., 2018; Marrugo-Ramírez et al., 2018; Siskova et al., 2020). This technology is commercialized as a kit called CellSearch, which is the only kit presently approved by the FDA as a method of detecting CTCs in patients with metastatic colorectal, breast, or prostate cancers (Cheung et al., 2018; Vafaei et al., 2020; Wu et al., 2020). The CellSearch kit allows detection of CTCs at a density of few tumor cells in whole blood sampling and represents high performance of 99 and 97% for specificity and sensitivity, respectively (Fernández-Lázaro et al., 2020). The limitation of this technique is described in Table 1. Besides the CellSearch kit, there are other tests based on the same principle, CellCollector, CellMax CMx, Cytelligen, AdnaTest, DEPArray, and others. All these tests allow the detection of CTCs to be monitored in the patients during treatment, with the main aim of either predicting recurrence of tumor or being used for downstream molecular profiling experiments (Shen et al., 2017).

## Liquid Biopsy Applications

### In Early Diagnosis and Screening of CRC

The most studied noninvasive tools for CRC detection are hemoccult test and fecal immunochemical test (FIT). Several years of assessment highlighted important limitations of these tools. Hemoccult test and FIT showed less than satisfactory performances and difficulties in the interpretation of results (Lieberman, 2010; Imperiale et al., 2014; Lou and Shaukat, 2021). To overcome those limitations, liquid biopsy has been investigated as an alternative for more than two decades and has shown consistent and satisfactory performance, thanks to the development of cutting-edge molecular biology instruments, which enable the analysis of released tumor traces ctDNA and CTC in the body fluids. ctDNA quantity and CTC numbers in body fluids are observed at high levels in colorectal cancer cases, especially at advanced stages. For instance, the mean concentration of ctDNA in blood or stool is 5–50 times higher in CRC patients than in healthy subjects (Pindler et al., 2015; Vymetalkova et al., 2018), and regarding CTC, CRC patients could contain 5–50 CTCs per few mL of whole blood (Gold et al., 2015; Pantel and Alix-Panabieres, 2017).

Recently, the detection of several tumor markers in ctDNA or in CTC has attracted considerable attention in CRC screening as well as in diagnosis. Detecting colorectal cancer at the early stage based on liquid biopsy is an effective strategy in reducing patient mortality and in increasing overall survival (OS) in affected patients. The use of noninvasive samples can be a valuable tool for unraveling the genetic and epigenetic abnormalities involved in tumorigenesis (Gold et al., 2015). The CRC molecular assays currently in use are based on the assessment of genetic or epigenetic modifications or both. The main gene alterations known to date are as follows.

Currently, mutations in the KRAS, BRAF, APC, and TP53 genes have been widely detected in the ctDNA of CRC patients. The analysis of the mutations found for these genes in tumor tissue and plasmatic ctDNA showed significant concordance, up to 100% (Vidal et al., 2017; Vymetalkova et al., 2018; Li et al., 2019). The **KRAS** gene is mutated in 35–45% of CRCs, with a high frequency in codons 12, 13, 59, 61, and 146. In advanced colorectal cancers, more than 50 different **BRAF** mutations have been documented for CRC, with 90% consisting of a change from thymine to single base adenine at position 1799, located in exon 15 and producing a substitution at codon 600 that replaces glutamine with valine (V600E; Wang et al., 2017). In addition to that, mutational inactivation of the **APC** gene was detected in about 85% of sporadic cases and frequently seen in distal rather than proximal colon cancer sites (Markowitz and Bertagnolli, 2009). Also, germline mutation of the APC gene was described and is inherited in familial adenomatous polyposis (FAP) patients (Powell et al., 1993). Mutation of the APC gene leads to the activation of the tumor suppressor genes DCC/DPC4 and TP53 and to the activation of oncogenes such as COX2 and KRAS (Markowitz and Bertagnolli, 2009). The **TP53** gene mutation has been widely detected in non-hypermutated colorectal cancers with a percentage of approximately 55–60% and represents the second abnormality observed after the APC mutation (Nakayama and Oshima, 2019). The majority of TP53 mutations occur at exons 4, 5, 6, 7, and 8, especially between codons 100 and 300 (Li et al., 2019). In addition to the previously described molecular alterations seen in CRC, epigenetic phenomena such as CpG island methylator phenotype (CIMP) are also involved in CRC carcinogenesis; this alteration accounts for nearly 10–40% of all sporadic cases (Freitas et al., 2018). As an example of the epigenetic modification, methylation of the promoter region of the **MLH1** gene is strongly linked to the so-called methylator phenotype; it is reported in 22–49% of CIMP-positive tumors (Levine et al., 2016). Our group and other researchers identified several noninvasive methylation biomarkers, which in combination have demonstrated their diagnostic effect in CRC detection, such as **WIF**, **NPY**, **PENK**, **SEPT9**, **VIM**, **ALX4**, and others (Amiot et al., 2014; Jung et al., 2020).

Measuring the genetic and epigenetic information of a combination of several regions of genomic DNA sequence is useful in developing sensitive and highly specific noninvasive tumor diagnosis tests for early-stage CRC detection. In clinical practice, the liquid biopsy tests developed for screening Use either genetic or epigenetic targets or a combination. These biomarkers have passed several clinical trial validations and have been used to build new classes of assays; some are already approved by the FDA or designed as breakthrough devices and can be found in the market. The main tests are the following:

- The Epi proColon test, also known as the mSEPT9 test, is the first blood test designed for the identification of SEPT9 methylation in patients with colorectal cancer. This test is based on the extraction of ctDNA in the plasma and the amplification of the methylated DNA fragments by PCR (deVos et al., 2009). The Epi proColon Kit was approved by the Chinese Food and Drug Administration and the United States FDA in 2015 and 2016, respectively. The SEPT9 methylation test has good detection sensitivity. Numerous studies compared the performance of the mSEPT9 test with the FIT test. Research carried out showed that the mSEPT9 test is more sensitive than the FIT test (Johnson et al., 2014; Jin et al., 2015; Wu et al., 2016). The sensitivity of mSEPT9 is slightly higher ranging from 73 to 77% against 58 to 74% for the FIT test. The specificity of the two tests is high reaching 94% for mSEPT9 and 97% for the FIT test (Johnson et al., 2014; Jin et al., 2015; Wu et al., 2016).
- The monoplex assay for methylated VIM is currently marketed as a laboratory-developed test under the name of ColoSure™. This test is a single-marker test used to identify methylation of Vimentin in colorectal cancer. The test was developed by the Laboratory Corporation of America (LabCorp; Ned et al., 2011). Through several clinical studies, aberrant methylation of VIM alone has been detected with good sensitivity and specificity in CRC and pre-cancerous adenomas (Ned et al., 2011). The ColoSure test shows good performance ranging from 72 to 83% and from 53 to 86% for sensitivity and specificity, respectively. In addition, the ColoSure test represents an alternative screening method recommended especially in patients who cannot use standard diagnosis methods such as colonoscopy (Ned et al., 2011; Hamzehzadeh et al., 2017).
- The Cologuard test, also known as the mt-sDNA screening test, is a technology that allows the detection of DNA biomarkers in fecal samples. The Cologuard Screening test was developed by EXACT SCIENCES Corporation and Mayo Clinic, approved by the FDA in 2014 as a CRC screening tool and recommended by the College of American Pathologists. This test is recommended for asymptomatic subjects with average or high risk to develop CRC. The Cologuard test allows the quantification of a panel of distinct biomarkers consisting of the mutated KRAS gene; the methylation of the DNA promoter regions of genes NDRG4, BMP3, VIM, and TFP12; and the assessment of the fecal occult blood (Imperiale et al., 2014). In a clinical trial that screened 9,989 subjects, the effectiveness of Cologuard was established. In comparison to the FIT test, the Cologuard test was shown to be more accurate in detecting cancers and advanced adenomas, 92 vs. 74% for CRCs and 42 vs. 24% for advanced adenomas. However, the specificity of Cologuard was lower than for FIT, and it gave a negative screening result for only 87% of the normal subjects, while FIT provided accurate negative results for 95% of these normal subjects (Imperiale et al., 2014).

### In CRC Prognosis

Liquid biopsy has other potential applications in clinical practice such as CRC patient’s follow-up. Among the biomolecules detected in body fluids, ctDNA has shown prognostic value in patients with metastatic colorectal cancer (mCRC; Pindler et al., 2015). Numerous studies have shown that an increased ctDNA level is associated with shorter OS in patients with mCRC, while patients with low plasma ctDNA levels are associated with a longer OS (Pindler et al., 2015; Shen et al., 2017; Osumi et al., 2019). More specifically, KRAS or BRAF mutations in ctDNA have a prognostic aspect in mCRC and are correlated with shorter progression-free survival (PFS) and OS (El Messaoudi et al., 2016; Shen et al., 2017). Besides ctDNA, several biomarkers, which allow the detection of CTC, are considered as prognostic markers in CRC, like CK20-positive markers, leading to shorter OS. Other biomarkers, such as CK19, CD133, GCC, EPCAM, SURVIVIN, MUC 1, MUC 2, and hTERT, have shown their prognostic effect in CRC (Vafaei et al., 2019). In recent years, some biomarkers resulting from methylation aberration have also shown their prognostic value in CRC, taking as an example the methylation of the following genes: TAC1, IGFBP3, CDKN2A (p16), SEPT9, HPP1, TFPA2E, EVL, HLTF, CD109, BNIP3, NRCAM, MLH1, MGMT, CDKN2A (p14), and APC (Ma et al., 2019). Despite that the literature approves that methylation biomarkers have a prognostic effect, those biomarkers are not clinically applicable yet, due to the lack of validation studies (Draht et al., 2018).

### Treatment Guidance and Post-treatment Monitoring

**Figure 2:**
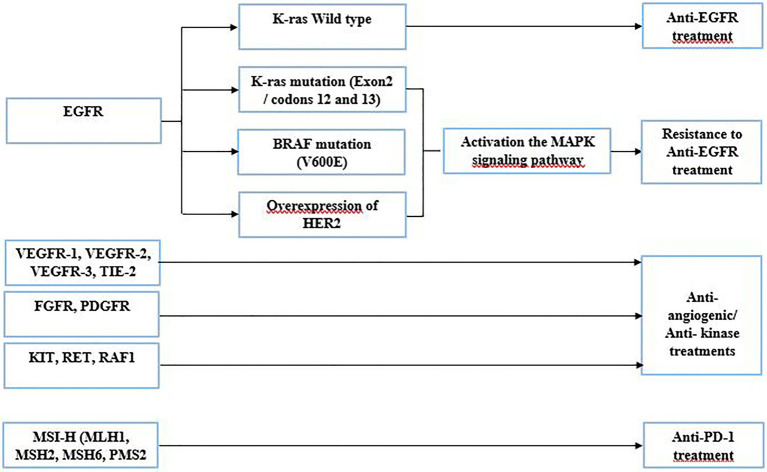
Gene alterations used in CRC treatment.

The identification of molecular anomalies associated with CRC has progressed in recent years. Liquid biopsy is a new strategy in performing a comprehensive tumor genomic profiling to aid for cancer treatment. Based upon genomic drivers of tumorigenesis, oncologists can make treatment recommendations and ensure the use of the right targeted therapy to the right patient. The use of targeted therapies has dramatically improved the OS of patients with mCRC (Martini et al., 2017). The detection of gene anomalies in liquid biopsy has been improved considerably; it constitutes a potential tool to identify actionable mutation, which could be targeted by a specific therapy (Martini et al., 2017). The following are the gene alterations used in CRC treatment (Figure 2):

- Patients harboring EGFR gene mutation, which constitutively activates the EGF receptor, could be treated with anti-EGFR drugs, such as cetuximab or panitumumab. In some cases, this strategy remains ineffective due to the presence of resistant mutant proteins, which act in the EGFR downstream signaling pathway. Indeed, ctDNA enables the early detection of possible resistance and guide for other treatment options (Ohhara et al., 2016; Martini et al., 2017). Cetuximab or panitumumab binds to EGFR, causing antitumor activity. In some cases, these drugs are not functional due to molecular aberrations in effectors of the EGFR pathway, such as mutations in exon 2 (codons 12 and 13) of the KRAS gene. These mutations involve activation of the MAPK pathway, thus leading to resistance to anti-EGFR treatment (Bronte et al., 2015). Before determining the targeted therapy, confirmation of the tumor status of the RAS gene is required and liquid biopsy can help in that respect as well.
- HER2 and EGFR share many downstream pathways, such as RAS/RAF/MEK and PI3K/AKT. Thus, amplification of the HER gene could confer resistance to anti-EGFR treatment (Xie et al., 2020). Also, it was described that PI3K mutations and PTEN loss might be associated with EGFR blockade resistance (Xie et al., 2020).
- BRAF gene mutations are also known to be an indicator of poor prognosis, and they are also identified as an adversely predictive biomarker of anti-EGFR treatment (Guo et al., 2019). Despite the blockage of EGFR, the BRAF mutation (V600E) provides resistance to treatment by activating the MAPK signaling pathway which promotes proliferation and survival of tumor cells. The resistance of the BRAF mutation to anti-EGFR therapy has been identified in several clinical trials (Zhao et al., 2017). These assays demonstrate a strong relationship between the presence of the BRAF mutation (V600E) and resistance to anti-EGFR therapy in colorectal cancer (Zhao et al., 2017).
- Several genes encoding protein kinases can be found frequently mutated in CRC with gain of function. Regorafenib is a multi-targeting inhibitor introduced in the clinical setting of mCRC (Arai et al., 2019), involving the blocking of aberrant activation of a panoply of protein kinases. These protein kinases are related either to the angiogenic pathway (VEGFR-1, VEGFR-2, VEGFR-3, and TIE-2) or to the oncogenic pathway (KIT, RET, RAF1, and BRAF). In two randomized phase III trials CORRECT and CONCUR, conducted in a population of patients with mCRC, regorafenib significantly increased OS and PFS with a reported benefit of 6.4 vs. 5.0 months and 1.9 vs. 1.7 months compared to placebo, respectively (Ohhara et al., 2016).
- Two other genes are found altered in CRC, the fibroblast growth factor receptor (FGFR) and the platelet-derived growth factor receptor (PDGFR). The aberrated FGFR form ends up behaving as an oncogene, playing a crucial role in the migration, proliferation, angiogenesis, and survival of cancer cells. Mutation of FGFRs leads to deregulation of downstream pathways leading to the formation of mitotic and anti-apoptotic cells (Cha et al., 2018). PDGFs are often mutated or overexpressed, leading to deregulation in the PDGF/PDGFR signaling pathway. This dysfunction is associated with angiogenesis, metastasis, invasion, and resistance to targeted therapies in CRC patients (Manzat Saplacan et al., 2017; García-Aranda and Redondo, 2019). PDGFR plays an important role in cell growth, cell division, and blood vessel formation in cancer. Regorafenib could be used to block the aberrant activity of the FGFR and the PDGFR (Bignucolo et al., 2017).
- Lastly, some CRC patients will have tumors that are microsatellite unstable, also known as microsatellite-high (MSI-H). The MSI-H phenotype is associated with germline defects in the following genes: MLH1, MSH2, MSH6, and PMS2. A programmed cell death protein 1 (PD-1) antibody is recommended in patients with microsatellite-unstable tumors (Overman et al., 2018; Oliveira et al., 2019).

Based on the genomic profile provided by the liquid biopsy test, oncologists can make treatment recommendations. The assessment of the genetic status of validated biomarkers in a noninvasive manner can be used as companion test for targeted therapies. These targeted therapies have shown to increase the quality of life of affected patients compared to standard chemotherapies. Moreover, the use of liquid biopsy is an effective tool in posttreatment monitoring and can provide a comprehensive genetic profile of tumor heterogeneity, which could change over time (Reece et al., 2019).

## Promising CRC Biomarkers

We performed a literature search to capture identified tumor-related markers in body fluids (blood, stool, and urine), reported to be used for screening, diagnosis, or monitoring of CRC patients. Thereby, the search yielded 60 relevant biomarkers. The output data are reviewed and summarized in Table S1. We identified several classes of alterations: gene mutation, gene methylation, and changes in RNA expression and protein levels. The performance of these tumor markers remains relatively encouraging ranging from 6 to 99% and 29 to 100% for sensitivity and specificity, respectively, but with a largely variable cohort size, ranging from 29 to 2,975 recruited subjects (Okugawa et al., 2015; Rodia et al., 2016). The development of noninvasive genetic assays raises considerable hope for the possibility of reducing CRC mortality. A promising alternative to gene methylation and mutation is miRNA expression, which can also be detected in both serum and stool. These molecules are particularly interesting, being small, much more resistant to degradation than coding RNA, and easily profiled in body fluids. In addition, in many studies they show good performance in distinguishing CRC-affected patients from healthy subjects.

## Technical Limitations and the Future of the Liquid Biopsy

The advantages of liquid biopsy are unquestionable; however, we should admit that in clinical routine, we have still been facing several limitations. These include the following: (1) contamination by fragmented constitutive DNA adds to the critical need for adequate storage/quick processing after sampling and that of special precaution having to be taken for sample preservation, by using a specific buffer to avoid cell lysis and DNA/RNA release from hematological cells. (2) Not all tumors are shedding CTC or ctDNA and the use of liquid biopsy for these patients is therefore useless. (3) Usually, ctDNA in cancer patients occurs in low amounts in the blood compared to cfDNA. This fact is due to several factors such as cancer stage, tumor vascularization, tumor burden, metastatic potential of cancer cells, and the rate of necrosis and apoptosis. (4) The small size of ctDNA fragments makes it difficult to identify large-scale DNA rearrangements. (5) Methylation anomalies are rarely investigated, because assays need DNA treatment by sodium bisulfite, which consumes large quantities of DNA. (6) The volume of blood to be collected is high, compared to most blood tests, with at least 7 ml of whole blood being required. (7) Several platforms can be used for liquid biopsy testing, but many of them rely on technologies which are prone to errors, especially for those who need pre-amplification steps. (8) Some tumor-specific mutations can be extremely low, as low as 0.01% of total cfDNA, which can make the detection of low-frequency variants particularly challenging. (9) The only technology able to detect unknown variants is the NGS; however, to be able to detect low-frequency variants, large and potentially costly base coverage of the targeted region, defined as the number of reads covering a base, is an important prerequisite for reliable variant detection from NGS data. (10) The identification of genomic alterations with unknown significance or unclear effect on the gene function makes the interpretation difficult. (11) The use of exosomes and CTCs in routine diagnostics is limited; only CTCs that maintain epithelial features can be detected by the EpCAM, excluding CTCs with other characteristics (Vacante et al., 2020). Regarding exosomes, one of the main challenges limiting the clinical use of these EV is the lack of standardization and consistency regarding their isolation methods, which requires difficult and laborious methods (Drula et al., 2020). In addition, the high cost of the equipment and reagents intended for the most used technology in bioscience, NGS hinders the routine use of liquid biopsy in the clinical laboratory. Moreover, there is the need of expert and experienced specialists for sample handling from EV extraction to library preparation and the need for elaborate bioinformatics pipelines for data treatment and analysis.

Liquid biopsy still resists to be used routinely in clinical setting for CRC patient management (Arneth, 2018). Currently in clinical practice, liquid biopsy is used as a supplement to the conventional method, specifically when the tissue biopsy cannot be performed and/or the patient is resistant to any invasive procedure. In the field of the diagnosis of CRC, we still rely on the gold standard which is tissue biopsy rather liquid biopsy, to evaluate accurately the tumor cells and their microenvironment (Arneth, 2018).

Overall, the usefulness of liquid biopsy in the management of colorectal cancer is limited, but these limitations may be overcome soon. Liquid biopsy may provide a better real-time understanding of the dynamic of tumor growth and its evolution, which will lead to counter cancer development and metastasis. In terms of CRC treatment, drug development is lagging far away from the huge quantity of genetic information generated by liquid biopsy. The synchronization will be a huge trigger for the next level in personalized medicine in CRC.

## Conclusion

Liquid biopsy is an emerging field in the management of colorectal cancer, whose relevance as a potential diagnostic, prognostic, monitoring, and therapeutic tool makes it a viable strategy in clinical practice of CRC patients. Liquid biopsy also has certain limitations, but they seem to be at the reach of near-future technological development.

## Author Contributions

HM planned and supervised the work. OM and HM wrote the paper. RI and AO reviewed and edited the paper. All authors contributed to the article and approved the submitted version.

### Conflict of Interest

RI and HM are founders and shareholders of OncoDiag, a company specialized in biomarkers for noninvasive cancer diagnostics. AO is a cofunder of Al Azhar Oncology Center.

The remaining author declares that the research was conducted in the absence of any commercial or financial relationships that could be construed as a potential conflict of interest.
