# Supplementary material for: Updates on Clinical Use of Liquid Biopsy in Colorectal Cancer Screening, Diagnosis, Follow-Up, and Treatment Guidance
Source: Front Cell Dev Biol. 2021 May 24;9:660924. doi: 10.3389/fcell.2021.660924 (PMC8213391; doi:10.3389/fcell.2021.660924)
Supplement: Supplementary file 1 [file Data_Sheet_1.docx]

| **Biomarker** | **Alteration** | **Sensitivity** | **Specificity** | **Use** |  | **Sample** | **Stage** | **Cases** | **Reference** |
| --- | --- | --- | --- | --- | --- | --- | --- | --- | --- |
| **ALX4** | Methylation | 83,30% | 70.00% | Diagnostic |  | Blood | I - II | 30 | PMID : [26216839](https://www.ncbi.nlm.nih.gov/pubmed/26216839)  (Okugawa et al., 2015) |
| **Vimentin** | Methylation | 72,50% | 86,90% | Diagnostic |  | Stool | I - II | 40 | PMID : [26216839](https://www.ncbi.nlm.nih.gov/pubmed/26216839)  Okugawa et al., 2015) |
| **Wif-1** | Methylation | 19,00% | 99,00% | Screening |  | Stool | Advanced | 90 | PMID: [25025467](https://www.ncbi.nlm.nih.gov/pubmed/25025467)  (Amiot et al., 2014) |
|  | Methylation | 33,00% | 99,00% | Screening |  | Blood | Advanced | 90 | PMID: [25025467](https://www.ncbi.nlm.nih.gov/pubmed/25025467)  (Amiot et al., 2014) |
|  | Methylation | 27,00% | 99,00% | Screening |  | Urine | Advanced | 90 | PMID: [25025467](https://www.ncbi.nlm.nih.gov/pubmed/25025467) (Amiot et al., 2014) |
| **NGFR** | Methylation | 51.00% | 84.00% | Diagnostic |  | Blood | I - II | 133 | PMID : [26216839](https://www.ncbi.nlm.nih.gov/pubmed/26216839)  (Okugawa et al., 2015) |
| **HPP1** | Methylation | 21,10% | 100.0% | Diagnostic |  | Blood | I - II | 38 | PMID : [26216839](https://www.ncbi.nlm.nih.gov/pubmed/26216839)  (Okugawa et al., 2015) |
| **MGMT** | Methylation | 48.00% | 73.00% | Diagnostic |  | Stool | I - II | 29 | PMID : [26216839](https://www.ncbi.nlm.nih.gov/pubmed/26216839) (Okugawa et al., 2015) |
| **CDKN2A** | Methylation | 31.00% | 84.00% | Diagnostic |  | Stool | I - II | 29 | PMID : [26216839](https://www.ncbi.nlm.nih.gov/pubmed/26216839)  (Okugawa et al., 2015) |
| **miR-106a** | miRNA | 74.00% | 44.40% | Diagnostic |  | Blood | I - IV | 100 | PMID : [26261602](https://www.ncbi.nlm.nih.gov/pubmed/26261602)  (Chen et al., 2015) |
| **miR-20a** | miRNA | 46.00% | 73.42% | Diagnostic |  | Blood | I - IV | 100 | PMID : [26261602](https://www.ncbi.nlm.nih.gov/pubmed/26261602)  (Chen et al., 2015) |
| **SFRP2** | Methylation | 63,10% | 90,10% | Screening |  | Blood | Early | 122 | PMID : 31962098 (Zhao et al., 2020) |
| **SDC2** | Methylation | 56,60% | 95,60% | Screening |  | Blood | Early | 122 | PMID : 31962098 (Zhao et al., 2020) |
| **C9orf50** | Methylation | 76.00% | 91.00% | Diagnostic |  | Blood | Early | 113 | PMID : 31727158 (Jensen et al., 2019) |
| **KCNQ5** | Methylation | 83.00% | 95.00% | Diagnostic |  | Blood | Early | 113 | PMID : 31727158 (Jensen et al., 2019) |
| **CLIP4** | Methylation | 77.00% | 99.00% | Diagnostic |  | Blood | Early | 113 | PMID : 31727158 (Jensen et al., 2019) |
| **CCSP2** | Mutation | 44,40% | 86,70% | Diagnostic |  | Blood | Early | 81 | PMID : 31179210) (Jeun et al., 2019) |
| **TIMP-1** | Expression | 52.00%-  85.00% | 60.00%-  95.00% | Diagnostic |  | Blood | ND | 819 | PMID : [30458003](https://www.ncbi.nlm.nih.gov/pubmed/30458003)  (Meng et al., 2018) |
| **SDC2** | Methylation | 89,10% | ND | Diagnostic |  | Stool | 0 - II | 245 | [PMID : 30876480](https://www.ncbi.nlm.nih.gov/pubmed/30876480)  (Han et al., 2019) |
| **CNPY2**  **isoform 2** | Mutation | 93,50% | 29,30% | Diagnostic |  | Blood | I | 430 | [PMID : 30070972](https://www.ncbi.nlm.nih.gov/pubmed/30070972)  (Peng et al., 2019) |
| **NEAT1-v1** | Mutation | 69.00% | 79.00% | Diagnostic Pronostic | / | Blood | I - IV | 100 | [PMID : 26552600](https://www.ncbi.nlm.nih.gov/pubmed/26552600)  (Wu et al., 2015) |
| **NEAT1-v2** | Mutation | 70.00% | 96.00% | Diagnostic Pronostic | / | Blood | I - IV | 100 | [PMID : 26552600](https://www.ncbi.nlm.nih.gov/pubmed/26552600)  (Wu et al., 2015) |
| **miR-24** | miRNA | 78.38 % | 83,85% | Diagnostic |  | Blood | I - IV | 223 | PMID : [26297223](https://www.ncbi.nlm.nih.gov/pubmed/26297223)  (Fang et al., 2015) |
| **miR-320a** | miRNA | 92.79 % | 73.08 % | Diagnostic |  | Blood | I - IV | 223 | PMID : [26297223](https://www.ncbi.nlm.nih.gov/pubmed/26297223)  (Fang et al., 2015) |
| **miR-423-5p** | miRNA | 91.89 % | 70.77 % | Diagnostic |  | Blood | I - IV | 223 | PMID : [26297223](https://www.ncbi.nlm.nih.gov/pubmed/26297223)  (Fang et al., 2015) |
| **AREG** | Mutation | 55.00% | 80.00% | Screening |  | Blood | I - II | 98 | [PMID : 31652396](https://www.ncbi.nlm.nih.gov/pubmed/31652396)  (Bhardwaj et al., 2020) |
| **KRT19** | Mutation | 44.00% | 80.00% | Screening |  | Blood | I - II | 98 | [PMID : 31652396](https://www.ncbi.nlm.nih.gov/pubmed/31652396)  (Bhardwaj et al., 2020) |
| **OPN** | Mutation | 42.00% | 80.00% | Screening |  | Blood | I - II | 98 | [PMID : 31652396](https://www.ncbi.nlm.nih.gov/pubmed/31652396)  (Bhardwaj et al., 2020) |
| **GATA5** | Mutation | 14.00% | 99.00% | Diagnostic |  | Blood | I | 154 | [PMID : 25538088](https://www.ncbi.nlm.nih.gov/pubmed/25538088)  (Melotte et al., 2015) |
| **FOXE1** | Mutation | 35.00% | 93.00% | Diagnostic |  | Blood | I | 154 | PMID : 25538088 (Melotte et al., 2015) |
| **SYNE1** | Mutation | 28.00% | 96.00% | Diagnostic |  | Blood | I | 154 | PMID : 25538088 (Melotte et al., 2015) |
| **NDRG4** | Methylation | 76.20% | 89.00% | Diagnostic |  | Stool | I - IV | 84 | PMID : [25663916](https://www.ncbi.nlm.nih.gov/pubmed/25663916)  (Xiao et al., 2015) |
|  | Methylation | 54.80% | 78.10% | Diagnostic |  | Blood | I - IV | 84 | PMID : [25663916](https://www.ncbi.nlm.nih.gov/pubmed/25663916)  (Xiao et al., 2015) |
|  | Methylation | 72.60% | 85,00% | Diagnostic |  | Urine | I - IV | 84 | PMID : [25663916](https://www.ncbi.nlm.nih.gov/pubmed/25663916)  (Xiao et al., 2015) |
| **SEPT9** | Methylation | 45.00% | ND | Diagnostic |  | Blood | I | 2975 | [PMID : 28102859](https://www.ncbi.nlm.nih.gov/pubmed/28102859)  (Nian et al., 2017) |
| **Cyr61** | Protein level | 75,50% | 69,80% | Diagnostic |  | Urine | 0 - IV | 148 | PMID : [30611902](https://www.ncbi.nlm.nih.gov/pubmed/30611902)  (Shimura et al., 2019) |
| **TFF3** | Protein level | 75,50% | 69,80% | Diagnostic |  | Urine | 0 - IV | 148 | PMID : [30611902](https://www.ncbi.nlm.nih.gov/pubmed/30611902)  (Shimura et al., 2019) |
| **APC** | Methylation | 06.00%  57.00% | 86.00%-  100.0% | Screening |  | Blood | I - IV | ____ | PMID : 29678513 (Worm, 2019) |
| **M2-PK** | enzymatic assay | 79.00% | 80.00% | Screening |  | Stool | ND | 407 | PMID : [25888768](https://www.ncbi.nlm.nih.gov/pubmed/25888768)  (Uppara et al., 2015) |
| **MMP-9** | Protein level | 89.30% | 91.20% | Diagnostic |  | Stool | ND | 28 | PMID : [26908323](https://www.ncbi.nlm.nih.gov/pubmed/26908323)  (Annaházi et al., 2016) |
| **COL3A1** | Protein level | 98.80% | 69.10% | Diagnostic Pronostic | / | Blood | I - III | 407 | PMID : [26741506](https://www.ncbi.nlm.nih.gov/pubmed/26741506)  (Wang et al., 2016b) |
| **AZGP1** | Protein level | 55.80% | 85.00% | Diagnostic |  | Blood | I - IV | 120 | PMID : [25561225](https://www.ncbi.nlm.nih.gov/pubmed/25561225)  (Xue et al., 2014) |
| **IL-6** | Protein level | 72.22% | 75.00% | Diagnostic Pronostic | / | Blood | ND | 72 | PMID : [26765465](https://www.ncbi.nlm.nih.gov/pubmed/26765465)  (Xu et al., 2016) |
| **RBP4** | Protein level | 74.90% | 81.70% | Diagnostic |  | Blood | ND | 402 | PMID : [29190912](https://www.ncbi.nlm.nih.gov/pubmed/29190912)  (Fei et al., 2017) |
| **THBS2** | Protein level | 64.90% | 87.10% | Diagnostic |  | Blood | ND | 402 | PMID : [29190912](https://www.ncbi.nlm.nih.gov/pubmed/29190912) (Fei et al., 2017) |
| **ECAD** | Methylation | 65.20% | 88.00% | Screening |  | Stool | Early | ____ | PMID : [32104546](https://www.ncbi.nlm.nih.gov/pubmed/32104546)  (Loktionov, 2020) |
| **FBN1** | Methylation | 72.00% | 93.30% | Screening |  | Stool | Early | ____ | PMID : [32104546](https://www.ncbi.nlm.nih.gov/pubmed/32104546) (Loktionov, 2020) |
| **HIC1** | Methylation | 42.30% | 98.00% | Screening |  | Stool | Early | ____ | PMID : [32104546](https://www.ncbi.nlm.nih.gov/pubmed/32104546)  (Loktionov, 2020) |
| **ING1b** | Methylation | 73.70% | 95.00% | Screening |  | Stool | Early | ____ | PMID : [32104546](https://www.ncbi.nlm.nih.gov/pubmed/32104546)  (Loktionov, 2020) |
| **ITGA4** | Methylation | 40.00% | 96.80% | Screening |  | Stool | Early | ____ | PMID : [32104546](https://www.ncbi.nlm.nih.gov/pubmed/32104546)  (Loktionov, 2020) |
| **RASSF2** | Methylation | 45.30% | 94.70% | Screening |  | Stool | Early | ____ | PMID : [32104546](https://www.ncbi.nlm.nih.gov/pubmed/32104546)  (Loktionov, 2020) |
| **BMP3** | Methylation | 51.00%-  84.00% | 90.00%100.0% | ND |  | Stool | ND | ____ | PMID : [32104546](https://www.ncbi.nlm.nih.gov/pubmed/32104546)  (Loktionov, 2020) |
| **miR-21** | miRNA | 86.05% | 81.08% | Diagnostic |  | Stool | I - IV | 40 | PMID : [27432735](https://www.ncbi.nlm.nih.gov/pubmed/27432735)  (Bastaminejad et al., 2017) |
|  | miRNA | 86.05% | 72.97% | Diagnostic |  | Blood | I - IV | 40 | PMID : [27432735](https://www.ncbi.nlm.nih.gov/pubmed/27432735)  (Bastaminejad et al., 2017) |
| **ITGB6** | Protein level | 69.80% | 100.0% | Diagnostic  Pronostic  Surveillance | /  / | Blood | I - IV | 60 | PMID : 30653264 (Bengs et al., 2019) |
| **IGFBP2 +**  **DKK3 +**  **PKM2** | Protein level | 73.00% | 95.00% | Detection |  | Blood | I - IV | 145 | PMID : [25793510](https://www.ncbi.nlm.nih.gov/pubmed/25793510)  (Fung et al., 2015) |
| **miR-139-3p** | miRNA | 96.60% | 97.80% | Diagnostic |  | Blood | I - IV | 117 | PMID : [28404964](https://www.ncbi.nlm.nih.gov/pubmed/28404964)  (Ng et al., 2017) |
| **ALU115** | DNA fragments | 69,23% | 99.09% | Diagnostic |  | Blood | I - IV | 205 | PMID: [25157833](https://www.ncbi.nlm.nih.gov/pubmed/25157833)  (Hao et al., 2014) |
| **ALU247/115** | DNA fragments | 73,08% | 97,27% | Diagnostic |  | Blood | I - IV | 205 | PMID: [25157833](https://www.ncbi.nlm.nih.gov/pubmed/25157833)  (Hao et al., 2014) |
| **TSPAN 8** | mRNA | 83.60% | 58.20% | Detection |  | Blood | I - III | 67 | PMID: 26993598 (Rodia et al., 2016) |
| **COL1A2** | mRNA | 73.10% | 59.70% | Detection |  | Blood | I - III | 67 | PMID: 26993598 (Rodia et al., 2016) |
| **LGALS4** | mRNA | 82.10% | 61.20% | Detection |  | Blood | I - III | 67 | PMID: 26993598 (Rodia et al., 2016) |
| **CEACAM6** | mRNA | 65.70% | 61.20% | Detection |  | Blood | I - III | 67 | PMID: 26993598 (Rodia et al., 2016) |

ND: not determined

**Table 1: Promising Biomarkers described in CRC patients**
